# Correlation Analysis of Umbilical Cord Blood Metabolic Phenotype and Inflammation in Patients with Gestational Diabetes Mellitus Complicated with Overweight and Obesity

**DOI:** 10.1155/2022/6072286

**Published:** 2022-05-13

**Authors:** Qiuling Chen, Wenxia Li, Yanxia Deng, Yongqi Li, Le Huang, Liping Zhao, Hua Li

**Affiliations:** Department of Obstetrics, Changsha Hospital for Maternal & Child Health Care Affiliated to Hunan Normal University, Changsha 417000, Hunan, China

## Abstract

**Background:**

Gestational diabetes mellitus (GDM) is a common metabolic disorder in pregnancy. The incidence rate is increasing year by year, which seriously threatens the safety of maternal and infant. Obesity is a vital factor in inducing GDM. Pregnant women with GDM account for a large proportion of overweight and obese pregnant women. Our study aimed to explore the potential mechanism of differential metabolites on inflammation and find the intervention and management methods for GDM in overweight and obese pregnant women.

**Methods:**

Umbilical cord blood samples and placenta were collected from normal weight pregnant women with GDM (control group) and overweight and obese pregnant women with GDM (obesity group) for a comparative study. Serum inflammatory factors IL-10, TNF-*α*, IL-6, lipopolysaccharide (LPS), and TLR4 expression were detected by ELISA. The expression levels of BCL-2 and caspase-3 were measured by Western blot. TUNEL staining was used to observe the apoptosis of placental villi. KEGG combined with metabolomics was used to compare the differences of metabolic maps between the two groups.

**Results:**

Compared with the control group, the level of anti-inflammatory factor IL-10 in the cord blood was decreased in the obesity group, while the levels of proinflammatory factors TNF-*α*, IL-6, and LPS were increased. In the placental tissues, the obesity group had higher concentrations of LPS, TLR4, and caspase-3 and lower concentration of BCL-2. Placental villi in the obesity group were more likely to undergo apoptosis than the control group. Correlation analysis showed that the above metabolite concentrations were negatively correlated with TNF-*α* or LPS.

**Conclusion:**

Metabolites could control obesity in the process of controlling the occurrence and development of inflammation.

## 1. Introduction

Gestational diabetes mellitus (GDM) is a complication of pregnancy unique to women. The incidence of pregnancy-induced hypertension, eclampsia, and other diseases in GDM pregnant women is increasing, and the possibility of excessive fetal growth is also increasing. Infants are also more likely to develop various neonatal diseases including hyperbilirubinemia, hypocalcemia, erythema, and respiratory distress syndrome [1]. It can be seen that GDM has serious short-term or long-term harm to the mother and her offspring.

The etiology of GDM has a strong epidemiological correlation with physiological changes during pregnancy, obesity, individual genes, fetal maternal GDM exposure, and other environmental factors [2]. Among these factors, obesity is considered to be the most variable and important risk factor associated with GDM [3, 4], which can increase the risk of adverse pregnancy outcomes in pregnant women and their offspring, and independently contribute to the occurrence and development of GDM in pregnant women [5]. The increasing obesity worldwide has led to an upward trend in the incidence of GDM and perinatal complications related to the disease. People have gradually realized that it is important to strengthen the attention and research related to obesity and GDM [6]. Therefore, it is imminent to reduce the prevalence of GDM in pregnant women through weight management.

Studies have shown that GDM may affect the incidence of diseases in offspring through cord blood. The offspring of GDM showed demethylation, which was related to the risk of autism spectrum disorder, type 1/2 diabetes, and other diseases [7, 8]. Furthermore, the content of most fatty acids in GDM pregnant women and GDM infants was lower than those in the healthy pregnant women group [9]. The above studies have shown that cord blood is an important bridge between mothers and fetuses. However, the phenotypic characteristics of the overall metabolic profile of cord blood still need to be further understood.

In the pregnancy complications of obesity and GDM, placental inflammation had been observed to play a central role in the fetal environment [10]. Some bacteria could create a local anti-inflammatory environment in the placenta and transform it into a maintenance effect on the GDM state. However, compared with normal GDM pregnant women, the content of related bacteria was reduced in the placenta of overweight and obese GDM pregnant women. The anti-inflammatory effect was reduced, and it was not conducive to the progress of the metabolism that can easily lead to a more unfavorable inflammatory phenotype [11]. The correlation between the metabolism and inflammation provides a feasible path for us to intervene overweight and obese GDM pregnant women.

This study intended to collect cord blood and placental tissue samples from normal weight, overweight, and obese pregnant women who were clinically diagnosed with GDM. Metabolomics was used to analyze the differences in cord blood metabolism profiles between the two groups and screen out significantly different metabolites. We combined the changes in the levels of inflammatory factors in cord blood to explore the potential correlation between different metabolites and inflammation, in order to provide a theoretical basis for the intervention and management of GDM in overweight and obese pregnant women.

## 2. Methods

### 2.1. Source and Grouping of Subjects

This study was approved by the Clinical Research Ethics Committee of Changsha Maternal and Child Health Hospital (Approval No.: 2021013). During the experiment, all subjects informed consent to the conduct of this experiment. The test procedure was in line with the Declaration of Helsinki.

A total of 16 pregnant women with normal weight and 7 overweight and obese GDM pregnant women were included in the study. The subjects were all pregnant women who attended Changsha Maternity and Child Health Hospital from May 2020 to June 2021. The experiment was set as the control group and obesity group (pregnant women who were overweight or obese) according to the BMI classification standard. The cord blood had been collected from 16 cases in the control group and 7 cases in the obesity group, 23 cases in total, 2 copies for each case. Placental tissues were collected, with 5 cases in each group and 10 cases in total. The clinical characteristics of the subjects are given in Table 1.

Inclusion criteria: age 25–40; BMI standard was defined as overweight or obesity; and using the diagnostic criteria of the International Association of Diabetes and Pregnancy Research Groups (IADPSG), oral glucose tolerance test (OGTT), and blood glucose level 0 h ≥ 5.1 mmol/L, 1 h ≥ 10.0 mmol/L, and 2 h ≥ 8.5 mmol/L.

Exclusion criteria: pre-GDM: glycosylated hemoglobin (HbA1c) was more than 6.5% or fasting blood glucose (FBG) was more than 7 mmol/L or 2 h blood glucose level (OGTT) was more than 11.1 mmol/L; multiple pregnancy; suffering from chronic diseases such as metabolic and gastrointestinal health (such as inflammatory bowel disease); and diagnosis or history of clotting disorders.

### 2.2. Enzyme-Linked Immunosorbent Assay (ELISA)

The whole blood sample was placed overnight at 4°C and centrifuged at 1000 g at 2–8°C for 15 min, and the supernatant was taken out for later use. 25 *μ*L each of the protein sample was tested and the diluted BSA standard (Saibao, Yancheng, China) was added to the enzyme-labeled reaction wells of IL-10 (KE00170, Proteintech, USA), TNF-*α* (KE00068, Proteintech, USA), IL-6 (KE00139, Proteintech, USA), and LPS (CSB-E09945 h, Wuhan Huamei Biological Engineering Co., Ltd., China) kits at the same time, and then, BCA was added to work liquid complete quantitative detection. Subsequently, the absorbance value of each sample and BSA standard was measured with a microplate reader in the range of 540–590 nm.

### 2.3. Western Blot

We took 3–5 mL sample and washed them with PBS. Then, RIPA solution was added to lyse and centrifuged to obtain serum or tissue supernatant. The BCA method was used to quantitatively detect the protein concentration, and then, appropriate amount of protein supernatant was added according to the protein quantitative results for electrophoresis separation. The diluted primary antibody TLR4 (19811-1-AP, 1 : 5000, Proteintech, USA) was added to serum, and the diluted primary antibody BCL-2 (12789-1-AP, 1 : 2000, Proteintech, USA), caspase-3 (#9661, 1 : 1000, CST, USA), and the internal reference protein *β*-actin (66009-1-Ig, 1 : 5000, Proteintech, USA) were added to the tissue supernatant and incubated overnight at 4°C. Then, the samples were incubated with HRP-labeled secondary antibodies HRP goat anti-mouse IgG (SA00001-1, 1 : 5000, Proteintech, USA) or HRP goat anti-rabbit IgG (SA00001-2, 1 : 5000, Proteintech, USA) for 1.5 h at room temperature. ECL was used for color development and exposure imaging, and Quantity One professional gray analysis software was used to observe the intensity of protein bands.

### 2.4. TUNEL

We followed the instructions of the TUNEL kit (40306ES50, Shanghai Yisheng Bio, China). After the two groups of placental tissues were sliced, they were washed with PBS (pH = 7.2–7.6, Abiowell, China) for 3 times, and equilibration buffer was added and placed at room temperature for 10–30 min. Subsequently, 50 *μ*L of TdT enzyme incubation buffer was added to the sectioned tissues and incubated at 37°C in the dark for 30 min. The nuclei were stained with DAPI working solution (Abiowell, China) for 10 min at the same temperature and rinsed with PBS. Buffered glycerol (Abiowell, China) was used to seal slices, and slices were observed under the ×400 field of view of the fluorescence microscope (BA410 T, Motic, China). If the nucleus was stained brown, it was a positive apoptotic cell.

### 2.5. Nontargeted Metabolite Analysis

Seven cases were selected from cord blood of 16 in the control group, and the LC-MC analysis was performed together with cord blood of 7 in the obesity group. After the sample was diluted with water and rethawed, it was centrifuged for 15 min. The supernatant was taken for testing, and the data were preprocessed as required. PLS-DA analysis was used to investigate the sample quality, and the metabolites were screened for differences in genes between groups. At the same time, MetaboAnalyst 4.0 in conjunction with the Kyoto Encyclopedia of Genes and Genomes (KEGG) and Human Metabolome Database (HMDB) were used to conduct enrichment pathway analysis of metabolites in the two sets of cord blood.

### 2.6. Statistical Analysis

GraphPad Prism 8.0 was used for statistical analysis and drawing of all data in this study. The measurement data conforming to the normal distribution were expressed as the mean ±  standard deviation, and the comparison of the means between the two groups was performed by the *t*-test. Spearman correlation analysis was applied to conduct correlation study. *P* < 0.05 indicated a statistically significant difference.

## 3. Results

### 3.1. Inflammatory Factor Levels in Cord Blood

**Figure 1:**
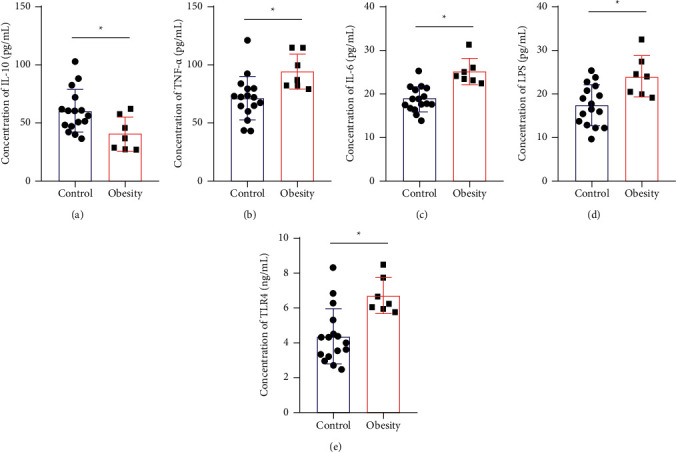
Inflammatory factor levels in cord blood. ELISA was used to detect the levels of IL-10 (a), TNF-*α* (b), IL-6 (c), LPS (d), and TLR4 (e) in cord blood of pregnant women with GDM. ^*∗*^*P* < 0.05 vs. the control group.

First, ELISA was used to detect the concentrations of anti-inflammatory factor IL-10 and proinflammatory factors TNF-*α*, IL-6, and LPS in cord blood of the two groups. The results of the measurement are shown in Figure 1. The level of IL-10 in the obesity group was much lower than that in the control group (Figure 1(a)), while the levels of TNF-*α*, IL-6, and LPS showed a significant upward trend (Figures 1(b), 1(c), and 1(d)). At the same time, the ELISA method was used to detect TLR4 expression, which showed that the content in the obesity group was significantly higher than that in the control group (Figure 1(e)). These results showed that the inflammatory factors and LPS in cord blood of the obesity group were released in large quantities, TLR4 was activated, and the proinflammatory response was triggered.

### 3.2. Changes in Umbilical Cord Blood Metabolism Atlas

**Figure 2:**
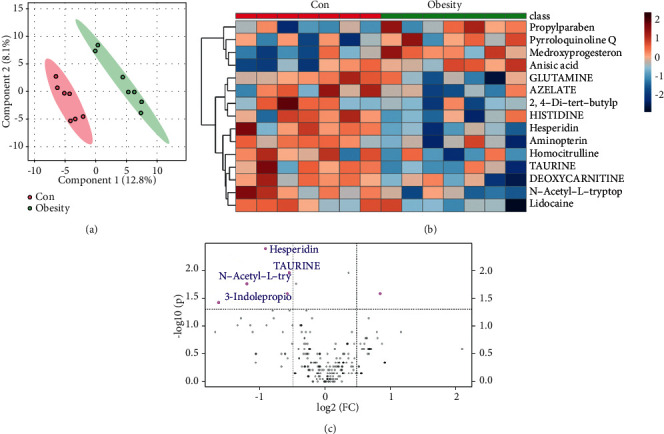
Changes in umbilical cord blood metabolism Atlas. (a) PLS-DA used to analyze the difference levels of metabolites between the two groups. (b) Heat map showing the enrichment of metabolites between the groups. (c) The volcano map showing metabolites with significant differences.

Next, nontargeted metabolic was applied to test cord blood of two groups. PLS-DA was used to analyze the differences in metabolites between the two groups: the respective regions of the two groups in the figure were clearly separated, and there were obvious differences in metabolomics components (Figure 2(a)). The results of the heat map analysis showed that compared with the control group, the obesity group had significantly higher levels of propylparaben, pyrroloquinoline Q, medroxyprogesterone, and anisic acid in cord blood. On the contrary, glutamine, azelate, histidine, and hesperidin and other metabolites levels were decreased (Figure 2(b)). *P* value was used to screen the metabolites with significant differences. The volcano chart clearly showed the upregulation or downregulation of metabolites: compared with the control group, the four metabolites of hesperidin, taurine, N-acetyl-L-tryptophan, and 3-indolepropionic acid were significantly downregulated in the obesity group, and their content was significantly reduced (Figure 2(c)).

### 3.3. Functional Changes of Differential Metabolites in Umbilical Cord Blood

**Figure 3:**
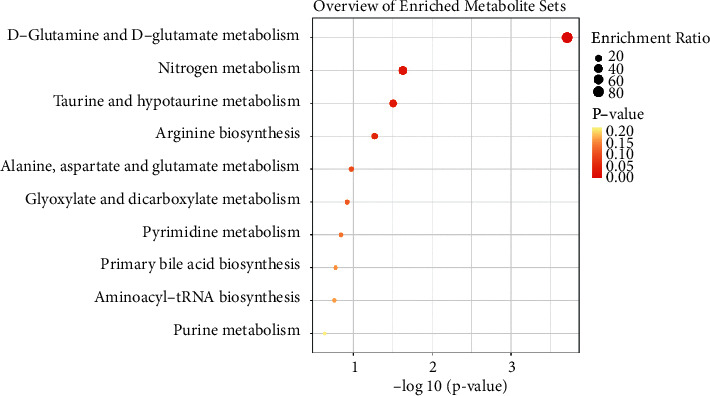
Functional changes of differential metabolites in cord blood. The enrichment analysis chart showed the main enrichment metabolic pathways of different metabolites.

The overview map of metabolome enrichment showed that metabolite enrichment was mainly concentrated in 5 metabolic pathways. Among them, the differential metabolites in the D-glutamine and D-glutamate metabolism pathways were the most enriched, followed by the nitrogen metabolism, taurine and hypotaurine metabolism, arginine biosynthesis, and alanine, aspartate, and glutamate metabolism (Figure 3).

### 3.4. Changes in Placental Tissue Inflammation

**Figure 4:**
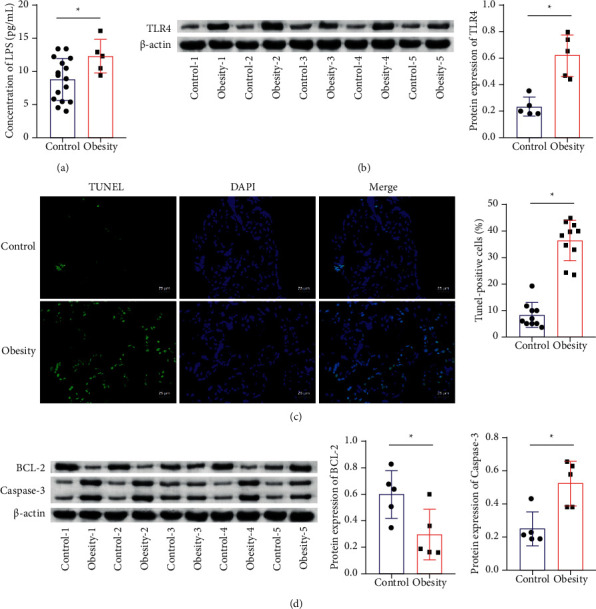
Changes in placental tissue inflammation. (a) ELISA applied to detect LPS concentration. (b) Western blot used to measure TLR4 content. (c) TUNEL staining performed to observe placental villi apoptosis (TUNEL, ×400, scale bar = 25 *μ*m). (d)-(e) Western blot applied to measure BCL-2 and caspase-3 proteins expression. ^*∗*^*P* < 0.05 vs. the control group.

The concentration of LPS in the placenta tissues was detected by ELISA, and the concentration of LPS in the obesity group was much higher than that in the control group (Figure 4(a)). Western blot was applied to detect the TLR4 level, which showed higher concentration in the obesity group (Figure 4(b)). TUNEL staining was used to observe placental villi apoptosis, which showed that the villi apoptosis of the obesity group was significantly increased than that of the control group (Figure 4(c)). At the same time, Western blot was applied to detect BCL-2 and caspase-3 levels. Compared with the control group, BCL-2 in the obesity group showed a lower level, but caspase-3 showed a higher level (Figures 4(d) and 4(e)).

### 3.5. Correlation between Inflammation and Changes in Metabolite Concentration

**Figure 5:**
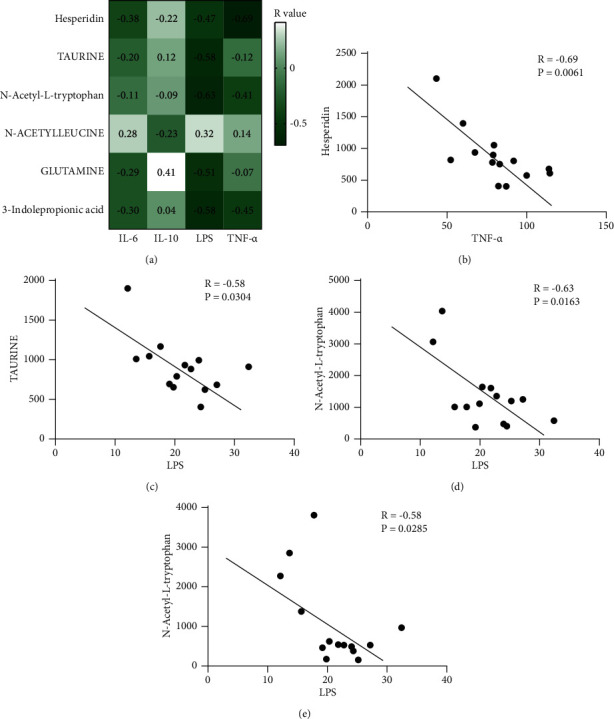
Correlation between inflammation and changes in metabolite concentration. (a) Correlation analysis between different metabolites and inflammatory factors. (b)–(e) The concentration of inflammatory factors varied with the content of metabolites. ^*∗*^Metabolites have a significant correlation with inflammatory factors.

We performed correlation analysis on the difference metabolites with significant changes in the content of the two groups in the above metabolic analysis and inflammatory factors. The change of hesperidin was significantly correlated with the content of proinflammatory factor TNF-*α*. Three metabolites of taurine, N-acetyl-L-tryptophan, and 3-indolepropionic acid were related to the change of LSP (Figure 5(a)). The reduction of the above four metabolites would lead to a significant increase in TNF-*α* or LPS (Figures 5(b)–5(e)). It could be considered that changes in the concentration of certain metabolites were indeed related to the occurrence of inflammation, and the lower the concentration of metabolites, the more likely inflammation will occur.

## 4. Discussion

The incidence of GDM in overweight and obese pregnant women is getting higher and higher, and it is an inevitable trend to continuously explore its treatment methods. GDM mostly reduces the risk from nutrient intervention and drug therapy [12, 13]. Studies have shown that women who supplemented with 600 *μ*g of folic acid before pregnancy can reduce the risk of GDM by about 30% than women who have not supplemented with folic acid [14]. Taking probiotics during pregnancy can effectively prevent GDM by regulating gut microbiota [15]. However, when overweight and obese women took probiotics during pregnancy, it was found that probiotics did not play a role in preventing GDM in overweight and obese pregnant women [16, 17]. It can be considered that exogenous nutrient intervention can reduce the risk of disease in GDM patients, but its effect on overweight and obese GDM pregnant women is unknown. The drug treatment may have an adverse effect on the offspring and lack long-term safety [12]. How to effectively intervene or treat GDM in overweight and obese pregnant women still needs further research.

Alan R. Saltiel et al. explored the inflammatory mechanism related to obesity and many metabolic diseases and confirmed the potential of anti-inflammatory therapy in the treatment of obesity-related diseases [18, 19]. The results of our study showed that the level of anti-inflammatory factor in the obesity group in cord blood and placental tissues was significantly lower than that in the control group, while the levels of various proinflammatory factors and LPS were significantly higher than those in the control group. We also verified that there was a certain correlation between obesity and inflammation, and the goal of controlling obesity could be achieved by inhibiting the occurrence and development of inflammation. The levels of related inflammatory factors had similar changes in cord blood and placental tissues. It could be considered that the mother's body affected the structure and function of the placenta through cord blood. Therefore, this study intended to reduce the prevalence of obese GDM mothers through changes in the content of differential metabolites in umbilical cord blood, while reducing the harm of GDM to the offspring.

The results of metabolomics analysis showed that hesperidin, taurine, N-acetyl-L-tryptophan, 3-indolepropionic acid, and other metabolites were significantly downregulated in the obesity group. Haijun Xiong et al. found that hesperidin can effectively alleviate the inflammation caused by hyperglycemia and hyperlipidemia and participate in the inhibition of adipogenesis to achieve the therapeutic effect on obesity [20, 21]. Tawar Qaradakhi et al. found that taurine is also involved in the process of the fat metabolism and has a good anti-inflammatory effect on cardiovascular diseases [22]. The metabolites of N-acetyl-L-tryptophan and 3-indolepropionic acid could alleviate or inhibit many inflammatory reactions [23]; 3-indolepropionic acid had been found to prevent steatohepatitis [24]. The above studies showed that four metabolites whose concentrations were significantly decreased in this differential metabolite analysis had a certain degree of the inflammation inhibitory effect, further illustrating that hesperidin and taurine were involved in the control process of obesity.

Our analysis of the correlation between differential metabolites and inflammation also showed that the above four metabolites were significantly related to TNF-*α* or LPS, and the lower the metabolite concentration, the more likely inflammation will occur. Hesperidin could improve colitis by reducing TNF-*α* [25], and taurine could slow down the occurrence of many diseases induced by LPS [26, 27]. However, the predecessors have not discussed the interaction mechanism of N-acetyl-L-tryptophan, 3-indolepropionic acid, and LPS. The above issues can be explored in the follow-up, and it is hoped that more breakthroughs in the treatment of inflammatory diseases can be achieved.

The above studies showed that we can mediate the occurrence and development of inflammation by regulating the content of related metabolites in overweight and obese GDM pregnant women, providing strong evidence for effective intervention and management of overweight and obese GDM. However, how to implement the theory in clinical operation and what kind of treatment effect will be obtained in the end still requires a lot of verification and practice by researchers. At the same time, due to the limitation of samples, we need to spend more time collecting samples of normal weight with GMD and overweight or obese with no GMD. The association between overweight and GDM was further confirmed by comparing the four groups: normal weight no GMD, normal weight with GMD, overweight or obese with no GMD, and overweight or obese with GMD.

In conclusion, metabolites controlled the occurrence and development of inflammation, so as to achieve the purpose of controlling obesity. This study explored the theoretical basis for intervention and management of overweight and obese GDM pregnant women from metabolomics.

## Figures and Tables

**Table 1:** Basic information of subjects.

	Control	Obesity	Significance
*n*	7	7	
Age (year)	30.43 ± 2.97	33.43 ± 3.77	ns
Height (cm)	157.57 ± 3.86	161.14 ± 3.27	ns
Prepregnancy weight (kg)	48.17 ± 1.99	77.07 ± 5.49	*∗*
Postpartum weight (kg)	58.74 ± 3.73	86.64 ± 9.66	*∗*
Prepregnancy BMI (%)	19.68 ± 1.28	29.65 ± 1.42	*∗*
Body fat percentage (%)	26.54% ± 2.24%	39.20% ± 3.49	*∗*
Fasting blood glucose (mmol/L)	4.49 ± 0.26	5.11 ± 0.39	*∗*
HbA1c (%)	4.97% ± 0.18%	5.48% ± 0.43%	*∗*

ns, no significance. ^*∗*^*P* < 0.05 vs. the control group.

## Data Availability

The data used to support the findings of this study are available from the corresponding author upon request.

## References

[1] Alfadhli E. M. (2015). Gestational diabetes mellitus. *Saudi Medical Journal*.

[2] Johns E. C., Denison F. C., Norman J. E., Reynolds R. M. (2018). Gestational diabetes mellitus: mechanisms, treatment, and complications. *Trends in Endocrinology and Metabolism*.

[3] Lende M., Rijhsinghani A. (2020). Gestational diabetes: overview with emphasis on medical management. *International Journal of Environmental Research and Public Health*.

[4] Farren M., Daly N., O’Higgins A. C., McKeating A., Maguire P. J., Turner M. J. (2015). The interplay between maternal obesity and gestational diabetes mellitus. *Journal of Perinatal Medicine*.

[5] Wang C., Wei Y., Zhang X. (2017). A randomized clinical trial of exercise during pregnancy to prevent gestational diabetes mellitus and improve pregnancy outcome in overweight and obese pregnant women. *American Journal of Obstetrics and Gynecology*.

[6] Schaefer-Graf U., Napoli A., Nolan C. J. (2018). Diabetes in pregnancy: a new decade of challenges ahead. *Diabetologia*.

[7] Howe C. G., Cox B., Fore R. (2020). Maternal gestational diabetes mellitus and newborn DNA methylation: findings from the pregnancy and childhood epigenetics consortium. *Diabetes Care*.

[8] Floris I., Descamps B., Vardeu A. (2015). Gestational diabetes mellitus impairs fetal endothelial cell functions through a mechanism involving microRNA-101 and histone methyltransferase enhancer of zester homolog-2. *Arteriosclerosis, Thrombosis, and Vascular Biology*.

[9] Ortega-Senovilla H., Schaefer-Graf U., Herrera E. (2020). Pregnant women with gestational diabetes and with well controlled glucose levels have decreased concentrations of individual fatty acids in maternal and cord serum. *Diabetologia*.

[10] Pantham P., Aye I. L., Powell T. L. (2015). Inflammation in maternal obesity and gestational diabetes mellitus. *Placenta*.

[11] Bassols J., Serino M., Carreras-Badosa G. (2016). Gestational diabetes is associated with changes in placental microbiota and microbiome. *Pediatric Research*.

[12] Szmuilowicz E. D., Josefson J. L., Metzger B. E. (2019). Gestational diabetes mellitus. *Endocrinology and Metabolism Clinics of North America*.

[13] Silva-Zolezzi I., Samuel T. M., Spieldenner J. (2017). Maternal nutrition: opportunities in the prevention of gestational diabetes. *Nutrition Reviews*.

[14] Li M., Li S., Chavarro J. E. (2019). Prepregnancy habitual intakes of total, supplemental, and food folate and risk of gestational diabetes mellitus: a prospective cohort study. *Diabetes Care*.

[15] Homayouni A., Bagheri N., Mohammad-Alizadeh-Charandabi S. (2020). Prevention of gestational diabetes mellitus (GDM) and probiotics: mechanism of action: a review. *Current Diabetes Reviews*.

[16] Callaway L. K., McIntyre H. D., Barrett H. L. (2019). Probiotics for the prevention of gestational diabetes mellitus in overweight and obese women: findings from the SPRING double-blind randomized controlled trial. *Diabetes Care*.

[17] Pellonperä O., Mokkala K., Houttu N. (2019). Efficacy of fish oil and/or probiotic intervention on the incidence of gestational diabetes mellitus in an at-risk group of overweight and obese women: a randomized, placebo-controlled, double-blind clinical trial. *Diabetes Care*.

[18] Gregor M. F., Hotamisligil G. S. (2011). Inflammatory mechanisms in obesity. *Annual Review of Immunology*.

[19] Saltiel A. R., Olefsky J. M. (2017). Inflammatory mechanisms linking obesity and metabolic disease. *Journal of Clinical Investigation*.

[20] Xiong H., Wang J., Ran Q. (2019). Hesperidin: a therapeutic agent for obesity. *Drug Design, Development and Therapy*.

[21] Tejada S., Pinya S., Martorell M. (2019). Potential anti-inflammatory effects of Hesperidin from the genus citrus. *Current Medicinal Chemistry*.

[22] Qaradakhi T., Gadanec L. K., McSweeney K. R., Abraham J. R., Apostolopoulos V., Zulli A. (2020). The anti-inflammatory effect of taurine on cardiovascular disease. *Nutrients*.

[23] Wang J., Yu S., Li J. (2019). Protective role of N-acetyl-l-tryptophan against hepatic ischemia-reperfusion injury via the RIP2/caspase-1/IL-1*β* signaling pathway. *Pharmaceutical Biology*.

[24] Zhang X., Coker O. O., Chu E. S. (2021). Dietary cholesterol drives fatty liver-associated liver cancer by modulating gut microbiota and metabolites. *Gut*.

[25] Zhang J., Lei H., Hu X., Dong W. (2020). Hesperetin ameliorates DSS-induced colitis by maintaining the epithelial barrier via blocking RIPK3/MLKL necroptosis signaling. *European Journal of Pharmacology*.

[26] Liu Y., Li F., Zhang L., Wu J., Wang Y., Yu H. (2017). Taurine alleviates lipopolysaccharide-induced liver injury by anti-inflammation and antioxidants in rats. *Molecular Medicine Reports*.

[27] Cheong S. H., Lee S. H., Jeon Y. J., Lee D. S. (2017). Mussel (*Mytilus coruscus*) water extract containing taurine prevents LPS-induced inflammatory responses in zebrafish model. *Advances in Experimental Medicine & Biology*.

